# Global Health Security Capacity and Capability Measurement Framework Within the Biological Threat Reduction Program

**DOI:** 10.1089/hs.2020.0023

**Published:** 2021-04-08

**Authors:** Nino Kharaishvili, Jane W. Blake, Douglas H. Gorsline, Lance R. Brooks

**Affiliations:** Nino Kharaishvili, MD, MBA, is Director, Global Health Solutions, Jacobs, Arlington, VA. Jane W. Blake, MS, is Lead Associate, Global Defense Group, Booz Allen Hamilton, Lorton, VA. Douglas H. Gorsline, MA, is Division Chief for INDOPACOM, Biological Threat Reduction Program, Defense Threat Reduction Agency, Department of Defense, Fort Belvoir, VA. Lance R. Brooks is Chief, Wide Area and Infrastructure Decontamination Branch, Office of Research and Development, US Environmental Protection Agency, Research Triangle Park. NC.

**Keywords:** Infectious diseases, Public health preparedness/response, Program evaluation, Biosafety, Biosecurity, Biosurveillance, Capacity development

## Abstract

The Biological Threat Reduction Program, part of the Nunn-Lugar Cooperative Threat Reduction Program since 1991, is mandated by the US Congress to regularly provide public reporting as part of its accountability. The Biological Threat Reduction Program recently designed a metrics and evaluation framework to measure its impact and effectiveness in partner countries. The framework focuses on capacity and capability strengthening related to biosafety, biosecurity, and biosurveillance. This is a marked shift from the previous approach, which relied on more tangible outcomes such as the elimination of weapons of mass destruction production assets, delivery devices, munitions, and construction activities. The new metrics and evaluation framework tracks the program's impact across 24 biosafety, biosecurity, and biosurveillance metrics and numerous capability, capacity, sustainability, and regional leadership indicators for human and animal health systems. The framework uses quantitative and qualitative inputs to generate measurement scores for program investment in partner countries. Overall, the framework provides a robust feedback loop between requirements, plans, and implementation processes throughout each step of the program's annual management lifecycle.

## Introduction

Founded in 1991 by US Senators Sam Nunn (D, Georgia) and Richard Lugar (R, Indiana), the Department of Defense Nunn-Lugar Cooperative Threat Reduction (CTR) Program was created with a clear and concrete goal in mind: to secure and dismantle weapons of mass destruction (WMD) and associated infrastructure in the former Soviet Union.^1^ The CTR Program aimed to secure or eliminate the large WMD arsenals and infrastructure inherited by former Soviet Union countries after the Soviet Union's collapse.^2^ In its early years, the program focused mostly on demilitarization of chemical and biological weapon production facilities, destruction of chemical munitions and nuclear missiles, and demolition of WMD delivery vehicles in the former Soviet Union partnering countries, including Armenia, Azerbaijan, Georgia, Kazakhstan, Russia, Ukraine, and Uzbekistan.^1,3^

**Figure 1.**
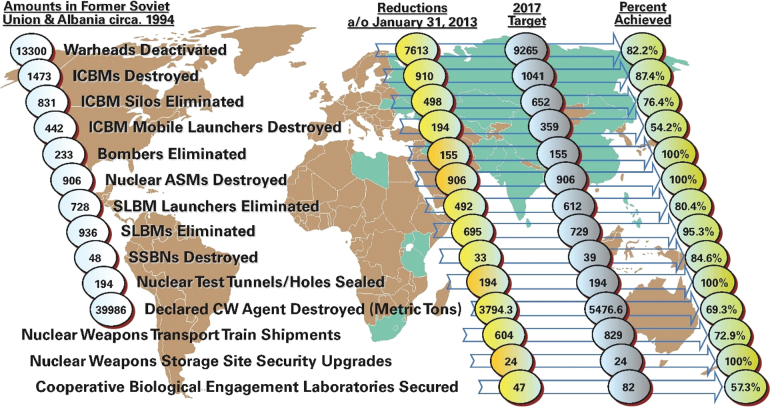
Cooperative Threat Reduction Program's 2013 metrics scorecard. Abbreviations: CW, chemical weapon; ICBM, intercontinental ballistic missile; SLBM, submarine-launched ballistic missile; SSBN, nuclear-powered, ballistic missile-carrying submarine. Color images are available online.

Since the inception of the CTR Program, Chapter 48 of US Code Title 50 requires that congressional investments in CTR demonstrate concrete evidence of threat reduction: “The Secretary of Defense shall implement metrics to measure the impact and effectiveness of activities of the Program to address threats arising from the proliferation of chemical, nuclear, and biological weapons and weapons related materials, technologies, and expertise.”^4^ To meet these requirements, the CTR Program, through its Biological Threat Reduction Program (BTRP), implemented a scorecard (Figure 1) to showcase programmatic metrics progress to its stakeholders.

The scorecard focused heavily on tangible outcomes such as the elimination of WMD production assets, delivery devices, munitions, and construction activities completed under the CTR Program. However, the BTRP information provided in the scorecard counted the number of facilities BTRP built and equipped but did not fully consider capabilities BTRP supported in conjunction with construction or its cooperative biological research program.*

This approach aligned well with the majority of CTR activities being conducted at the time and provided simple and reliably quantitative measurements, wherein the number of destroyed missiles, bombers, submarines, or facilities constructed clearly demonstrated the CTR Program's threat reduction value to the US Congress and the American public.^1^

With the conclusion of the former Soviet Union's demilitarization^3^ and a changing geopolitical environment, the CTR Program evolved and expanded into a broader countering WMD mission through implementation of capacity and capability strengthening programs around the globe. Over the years, BTRP has evolved from a narrowly scoped bioweapon infrastructure elimination program to a broader national security program, focusing on reducing natural, accidental, or intentional outbreaks that can cause potential harm to the United States or its allies.^6^ The program now aims to enhance biosurveillance^†^ and biosafety^‡^ and biosecurity^§^ systems in partner countries around the world. BTRP's desired end state is that partner countries have effective and sustainable biological threat mitigation systems that meet recognized international standards. BTRP's current mission is to improve the ability of partner countries to:^7^

1. Rapidly and accurately survey, detect, diagnose, and report biological terrorism and outbreaks of pathogens and diseases of security concern**
2. Identify, consolidate, and secure collections of especially dangerous pathogens to prevent the sale, theft, diversion, or accidental release of such pathogens

BTRP's evolved mission focuses exclusively on the less prevalent but more impactful group of especially dangerous pathogens (as defined by the US Select Agent List), whereas other US government offices predominantly work on more commonly occurring pathogens in partner countries.

In contrast to the original CTR scorecard, BTRP's shift from dismantling and destroying bioweapon infrastructure and stockpiles to improving global health security capacities and capabilities presented unique challenges for measuring and communicating the program's impact in partner countries. BTRP may have had occasional case studies that clearly demonstrated the program's impact on 1 or more discrete objectives at a point in time, the program impact on a larger scale was inconsistently measurable. A new metrics framework was needed to more accurately measure the progress and impact of BTRP's national security mission, which is focused on complex capacity and capability strengthening components.

## Methods

As early as 2009, the RAND Corporation recommended that the WMD community develop a common framework to assess the effectiveness of strengthening partner capacity programs and noted that assessing capability and capacity is difficult.^9^ Recognizing that the original CTR scorecard methodology no longer applied to its diversified programmatic activities, BTRP embarked upon a 4-phased approach to develop a revised programmatic measurement framework.

### Phase 1: Obtain Third-Party Recommendations

BTRP commissioned the National Academy of Sciences (NAS) in 2012 to review its evolved mission and metrics process to make recommendations on rectifying them. NAS published a 2012 report, *Improving Metrics for the Department of Defense Cooperative Threat Reduction Program*,^10^ which found that the program had reasonable metrics for consolidating and eliminating WMD but was unprepared to measure the newly added capacity and capability strengthening components. NAS recommended that BTRP shift their focus from the numbers of activities implemented, people trained, or facilities constructed, and instead focus on measuring biological threat reduction capabilities and capacities that demonstrate what countries could do after engaging with BTRP that they could not do before.

To develop the NAS recommendations into a regular, repeatable, and reliable methodology for the program, BTRP commissioned RAND to provide suggestions for an initial metrics framework. In addition to the NAS recommendations, RAND's 2014 report on *Measuring Cooperative Biological Engagement Program (CBEP) Performance*^11^ relied on extensive interviews with BTRP personnel to develop an initial logic-based metrics framework for the BTRP domains: biosecurity, biosafety, and biosurveillance. For each domain, RAND identified and characterized potential BTRP metrics according to validity, feasibility, utility, reliability, and relevance. Overall, RAND identified 73 potential metrics and further divided BTRP domains into subelements, with capacity and capability rating schemes to quantify program efficacy.

### Phase 2: Develop Guiding Principles

Upon receiving the recommendations, BTRP embarked on analyzing suggested metrics and evaluating them for the feasibility of implementation. The program's goal was to develop and implement a multitier metrics system to measure and report the program's progress and impact in partner countries. To achieve this end, BTRP established the following guiding principles and parameters for finalizing the metrics framework:

- Use a tiered approach to information analysis and reporting to address decision making needs of multiple levels of stakeholders, such as US Congress, Defense Threat Reduction Agency (DTRA) and CTR leadership, BTRP division chief, and BTRP project officers
- Address only BTRP's scope, mission, and engagement realities
- Make it measurable and quantifiable to the maximum extent possible
- Use a standardized and repeatable approach
- Use data that are feasible to obtain
- Make it practical to implement

### Phase 3: Evaluate and Adapt

BTRP used the guiding principles to review, evaluate, and adapt the recommendations from NAS and RAND. The program then used qualitative methods, such as focus groups, document analysis, and facilitated workshops, to complete the process of developing the metrics framework. BTRP focus groups included BTRP leadership, interdisciplinary subject matter experts, and other relevant stakeholders. The BTRP mission and scope dictated the composition of the subject matter expert focus groups, which included medical doctors, epidemiologists, laboratorians, veterinarians, health information technologists, and biosafety and biosecurity experts.

To evaluate the recommendations, the focus groups analyzed a set of relevant, discipline-specific metrics data against the guiding principles and evaluated the feasibility and applicability of each metric. BTRP consolidated individual evaluations and recommendations into a single change log for tracking and further adjudication, which BTRP presented to a broader subject matter expert panel to finalize the metrics framework. Two notable examples of metrics evaluation are described in Table 1.

**Table 1.** Metric Evaluation Examples

Initial Metrics (NAS and RAND)			BTRP Guiding Principle Alignment (High, Medium, Low)		Final Disposition		
Metric Category	Capability/Capacity/Sustainability	Metric Description	Feasibility	Applicability to Congressional Metrics	Final Disposition	BTRP Line of Effort	Rationale for Decline
Epidemiological analysis and investigation (Capacity)	Capability	Epidemiological investigation: Performance documented in written report or tested via exercise (tabletop or functional)	High	High	Indicator	Epidemiological analysis and investigation	N/A
Epidemiological analysis and investigation	Capability	# of suspected priority pathogen cases or outbreaks in past 12 months % for which an investigation was conducted and results documented	Low	Low	Declined	N/A	Data difficult to collect or inaccurate; may penalize countries with fewer outbreaks

Abbreviations: BTRP, Biological Threat Reduction Program; N/A, not applicable; NAS, National Academy of Sciences; RAND, RAND Corporation.

Once BTRP finalized the list of programmatic metrics, the subject matter experts developed a set of supportive indicators for each metric. They conducted further stakeholder interviews and document analysis to identify measurable and repeatable indicators that aligned with the programmatic metrics. Finally, they developed a proposed measurement scale and a descriptive methodology for each metric. Throughout development, BTRP conducted multiple peer reviews to seek external validation of the approach, including input from experts at the Walter Reed Army Institute of Research, the US Centers for Disease Control and Prevention, and Sandia National Laboratories.

### Phase 4: Refine and Finalize

To further refine and operationalize the measurement approaches, BTRP revised the metrics framework on 2 separate occasions. The first revision occurred in 2014, when BTRP conducted country capability assessments to collect real-time data from 2 partner countries. This effort resulted in minor adjustments to the metrics framework. BTRP then conducted a second and more extensive metrics framework refinement and validation by conducting assessments of 21 countries and integrating lessons learned as further adjustments to the metrics methodology.^12^

## Results

As noted in Table 2, the BTRP metrics framework measures the program's impact across 2 main pillars: (1) biosurveillance and (2) biosafety and biosecurity. BTRP subdivided the pillars into 7 associated lines of effort, 24 programmatic metrics, and 170 capability, capacity, sustainability, and leadership indicators. BTRP measures the pillars separately for human and animal health sectors. BTRP's Cooperative Biological Research Project supports all lines of effort in both sectors.

**Table 2.** Biological Threat Reduction Program Pillars, Lines of Effort, and Example Indicators

Pillar	Line of Effort	Example Programmatic Metrics	Example Indicators
Biosafety and biosecurity	Consolidating and securing pathogens	Control of pathogens of security concern is adequately maintained	An up-to-date biological agent and toxin inventory exists
	Facility-level biorisk management systems and culture	Necessary PPE is available, selected, and used properly	PPE is used appropriately
	National-level regulatory frameworks	National-level regulations, guidelines, or policies for BS&S are in place, are followed, and there are mechanisms for oversight and enforcement for BS&S regulations and/or guidelines	Regulations and/or guidelines for BS&S exists
Biosurveillance	Disease detection	Health professionals are trained in and demonstrate relevant clinical skills for disease detection	Point-of-care healthcare professionals are trained on recognition of pathogens and diseases of security concern and/or other disease detection skills
	Laboratory diagnostics	Equipment and reagents are available in country to permit effective and rapid detection of priority diseases	Laboratories have the necessary equipment to support reliable detection of pathogens
	Epidemiological analysis and investigation	Analysis of disease surveillance data is conducted to identify disease-specific trends and derive baseline data	Data are collected from all relevant jurisdictions within the country
	Reporting and communication	Reporting duties and reporting chain within the country are documented, effective, and used on a consistent basis by individuals from all components to support reporting to international organizations	Reports are sent to the national level from all districts

Abbreviations: BS&S, biosafety and biosecurity; PPE, personal protective equipment.

The biosafety and biosecurity pillar includes 3 lines of effort: (1) national-level regulatory frameworks, (2) consolidating and securing pathogens, and (3) facility-level biorisk management systems and culture. The biosurveillance pillar includes 4 lines of effort: (1) disease detection, (2) laboratory diagnostics, (3) epidemiological analysis and investigation, and (4) reporting and communications. All programmatic metrics and indicators measured within the 2 pillars focus exclusively on BTRP's congressionally mandated mission. For example, indicators associated with disease detection do not cover medical treatment components, as patient treatment is outside of BTRP's mission.

As noted in Table 3, BTRP measures progress on a scale of 1 to 5. Pillar scores are aggregated from line of effort scores, which are aggregated from programmatic metric subscores. Full descriptions of the pillar metrics by line of effort are located in Table 4 and Table 5.

**Table 3.** Biological Threat Reduction Program Metrics Scale

Score	Level	Description
1	Limited capacity	Partner country has basic capacity in place (eg, basic infrastructure, equipment, or documentation exists to perform a function).
2	Developed capacity	Partner country possesses all major capacity enablers. Relevant infrastructure and equipment are present to perform a function. In addition, pertinent standard operating procedures, guidelines, and training materials are developed and present.
3	Demonstrated capability	Partner country consistently demonstrates the use of tools, equipment, and material to perform a function. The trained personnel have skills and techniques to successfully operate in their respective fields.
4	Sustained capability	Partner country plans and implements processes and procedures that enable sustainability of provided capacity and capability.
5	Regional leadership/advanced capability	Partner country leads regional activities in a field by sharing expertise and other applicable tools to other countries.

**Table 4.** Biological Threat Reduction Program Biosafety and Biosecurity Lines of Effort and Descriptions

Score/Level	Consolidating and Securing Pathogens	Facility-Level Biorisk Management Systems and Culture	National-Level Regulatory Frameworks
1 - Limited capacity	Partner country has not considered consolidating collections of EDPs. Partner country does not have credible documentation on the presence or absence of those collections.	Critical gaps have been identified in BRM in many facilities across the partner country. General lack of awareness among the laboratory workforce of international best practices for safe, secure, and responsible conduct.	Partner country has not identified the need to establish regulations and/or guidelines for BS&S and has shown no or limited interest in compliance with international best practices.
2 - Developed capacity	Partner country is willing to consolidate collections of pathogens of security concern in principle but has not yet taken action. Partner country has adequately documented the presence of those collections.	Partner country has implemented some elements of BRM in some facilities. Laboratory workforce has general awareness of international best practices for safe, secure, and responsible conduct.	Partner country has identified the need to establish regulations and/or guidelines for BS&S, understands the importance of compliance with international best practices, and has initiated development of a national framework.
3 - Demonstrated capability	Partner country has made progress toward consolidating collections of pathogens of security concern. Partner country maintains accurate inventory records and demonstrates best practices for material accountability for those collections.	Partner country has implemented most elements of BRM at most facilities. Laboratory workforce demonstrates proficiency in applying international best practices for safe, secure, and responsible conduct.	Partner country has drafted regulations and/or guidelines for BS&S and is in the process of establishing a legal framework that includes mechanisms for oversight, enforcement, and attribution.
4 - Sustained capability	Partner country has consolidated collections of pathogens of security concern to a minimum acceptable level. Partner country has assessed and continually monitors the biorisks and vulnerabilities associated with the collections and actively addresses those risks to keep those collections secure.	Partner country has implemented and is self-sustaining BRM systems effectively at laboratory facilities throughout the country. Laboratory workforce demonstrates proficiency in applying international best practices for safe, secure, and responsible conduct and with a sustainable process in place for ensuring the continuation of training and maintenance of skills.	Partner country has regulations and/or guidelines for BS&S with fully implemented, self-sustained mechanisms for oversight, enforcement, and attribution.
5 - Regional leadership/ advanced capability	Partner country serves as regional leader by promoting the benefits of consolidation from a biosecurity and biosafety perspective and supports the use of qualified reference laboratories to minimize the number of containment laboratories throughout the region.	Partner country serves as regional leader by offering training, workshops, and other consultation on implementation of BRM systems and development of competent laboratory workforces for safe, secure, and responsible conduct to other countries in the region.	Partner country serves as regional leader by sustained and successful implementation of their regulatory framework to include sharing of lessons learned and promoting development of similar guidelines and legislation throughout the region.

Abbreviations: BRM, biorisk management; BS&S, biosafety and biosecurity; BTRP, Biological Threat Reduction Program; EDPs, especially dangerous pathogens.

**Table 5.** Biological Threat Reduction Program Biosurveillance Lines of Effort and Descriptions

Score/Level	Disease Detection	Laboratory Diagnostics	Epidemiological Analysis and Investigation	Reporting and Communication
1 - Limited capacity	No, or limited, ability to detect and diagnose EDPs within the partner country.	No, or minimal, laboratory diagnostic capability exists within the partner country.	No, or limited, coordinated ability exists to analyze surveillance data and investigate potential outbreaks of EDPs within the partner country.	No, or minimal, reporting network exists within the partner country.
2 - Developed capacity	Partner country has the capacity to detect and diagnose syndromes or EDPs at priority points of care.	Partner country is proficient in classical diagnostic techniques including bacteriology and serology in select laboratories but has limited referral and confirmatory processes.	Trained national and/or regional epidemiologists in the partner country have the required capacity to analyze data and investigate potential outbreaks of EDPs.	Partner country is in the process of developing and establishing protocols, processes, regulations, and/or legislation governing reporting and multisectoral coordination in response to biological events.
3 - Demonstrated capability	Partner country has the demonstrated capability to detect and diagnose EDPs at priority points of care.	Partner country has a national system of sample referral and confirmatory diagnostics culminating in performance of modern molecular or serological techniques at national and/or regional laboratories.	National and/or regional epidemiologists in the partner country have demonstrated their capability to collect and analyze integrated surveillance data on a weekly basis and have made progress toward establishing baselines for disease incidence.	Partner country demonstrates timely reporting of EDPs in alignment with national and international standards in select districts or regions.
4 - Sustained capability	Partner country has demonstrated the ability to detect and diagnose EDPs; there is a sustainable process in place for ensuring maintenance of skills and continuation of trainings.	Partner country has sustainable capability for performing modern molecular and serological techniques as part of a national system of sample referral and confirmatory diagnostics. Partner country is also able to leverage regional partnerships where needed to supplement the national laboratory network.	National and/or regional epidemiologists in the partner country conduct data analysis and share findings with other epidemiologists or those involved in outbreak investigation. A sustainable process is in place for ensuring continuation of trainings and maintenance of skills; baseline disease data are used for action at the district level.	Partner country demonstrates timely reporting of EDPs from district to international level; partner country has a sustainable process for maintaining and improving reporting, and communication capabilities and communication mechanisms are backed by established documentation (eg, protocols, regulations, legislation).
5 - Regional leadership/ advanced capability	Partner country has consistently demonstrated the ability to detect and diagnose pathogens/diseases related to those of security concern and has the ability to adapt disease detection capabilities in response to changes in the environment.	Partner country serves as a regional leader in performing modern diagnostic techniques when needed for the region. Partner country continues to demonstrate sustainable operations.	Partner country serves as a regional leader in performing epidemiological analysis and investigation when needed for the region. Partner country continues to integrate sustainment into training and other activities. Partner country shares epidemiological knowledge with neighboring countries.	Partner country serves as a regional leader in timely reporting of outbreaks involving EDPs and leads efforts to exercise regional capabilities.

Abbreviations: BSV, biosurveillance; BTRP, Biological Threat Reduction Program; EDPs, especially dangerous pathogens.

Finally, the BTRP metrics framework calculates 4 distinct measurement scores for each pillar, line of effort, and programmatic metric: start, current, threshold, and objective. The start score measures conditions before BTRP engagement with partner countries; the current score measures scores during period metrics reviews (no less than 1 per year); the threshold score is the realistic “target” determined by BTRP based on actual plan and available funding; and the objective score is the potential score that could be achieved in 5 years based on a country's technical capabilities, with no funding, legal, or political/cooperation constraints. Calculations for each score are generated from the 170 capacity, capability, regional leadership, and sustainability indicators. BTRP uses all 4 scores to inform requirements generation and key planning documents. BTRP does not publicly share the annual review scores. However, the program encourages partner countries to use relevant BTRP data in their decision-making process for improving capabilities in biosurveillance, biosafety, and biosecurity.

## Discussion

BTRP launched the program-wide metrics framework implementation in 2016 and has conducted annual reviews of metrics performance since 2017. Annual metrics measurements occur through internal desktop reviews or in-country operational evaluations performed by third parties. Annual measurements capture progression and regression along the 5-point scale and result in programmatic course corrections and provide accountability to Congress. To date, the metrics framework demonstrates a reliable and repeatable ability to measure program progress and determine capability maturation. Overall, BTRP is achieving expected incremental progress in strengthening capacity and capability across the 2 pillars in most partner countries and uses metric scores to identify lines of effort that require a more targeted focus. In addition, the system captures not only progress but also regression due to political or other external factors, thereby enabling course correction.

The metrics framework development and implementation process has had a positive impact well beyond BTRP's ability to measure progress. For example, the approach has triggered widespread refinements to BTRP's internal program acquisitions and planning processes. As a result, the metrics framework now underpins most components of BTRP's annual management cycle, provides a baseline for the entire program, and ensures that BTRP measures progress in each partner country using standardized language, pillars, lines of effort, and indicators. The metrics also support every programmatic requirement development in BTRP's individual country project plans and cooperative biological research projects. Lastly, BTRP uses the metrics framework to structure its approaches to internal program management reviews. Consequently, BTRP now has a functioning, integrated annual management cycle, in which the metrics framework plays a key role by providing a consistent feedback loop between programmatic requirements, project plans, and the implementation process throughout each step of the cycle.

By integrating the lessons learned from BTRP, other CTR programs are in the process of updating their own metrics frameworks. Streamlining and standardizing an impact measurement process across CTR programs ensures common reporting protocols within DTRA and enables data analysis and harmonization in reporting to the US Congress. By building upon the BTRP metrics framework and lessons learned, partner CTR programs can more rapidly develop and implement their own metrics frameworks.

Externally, BTRP is becoming known for its robust metrics framework and expertise. The program's efforts have both influenced and strengthened collaborative efforts with interagency partners. For example, BTRP recently worked with US Centers for Disease Control and Prevention and the United States Agency for International Development to develop an interagency framework for reporting on US government contributions to the Global Health Security Agenda. The goal of the effort is to deconflict measurement tools and techniques across the interagency, reduce redundancies, and address reporting gaps with the purpose of drafting an all-encompassing metrics framework across the US government for measuring Global Health Security Agenda progress within partner countries across the US government. In addition, BTRP's framework complements and contributes to Joint External Evaluations.^13^

Developing and implementing a robust new metrics framework comes with challenges. First, the metrics framework has significant technical requirements including data storage, validation, and analysis. Second, integrating legacy engagements or new types of future engagements into the framework requires further development. Third, large-scale implementation across all BTRP engagements requires organizational culture shifts, with associated change management considerations, including gaining adoption and buy-in from the program managers. Finally, while the framework measures the program's progress in an individual country, the framework does not yet consistently measure more complex regional impact nor return on relationships.^13,14^

During the development of this article in 2020, NAS published a report entitled *A Strategic Vision for Biological Threat Reduction: The U.S. Department of Defense and Beyond*.^6^ The report noted that BTRP has a complex mission that relies on coordinating with many partners domestically and internationally. The program is also challenged with ongoing revolutions in the life sciences, ease of access to information, rapid transportation of people, and widespread trade in animals and plants. All point toward novel threats from new actors, shorter timelines, and less geographic protection. The NAS report discusses the following key areas for a 5-year vision: embrace an integrated view of biological threats, identify needs and opportunities, select international partners, select partners in the United States, strengthen relationships and build networks within the department of defense, and evaluate progress and refine approaches. Engaging new partners or expanding partnerships with other implementation programs requires a good understanding of where impact can be made in partner countries. This will require further analysis to provide a common operating picture to help determine where the most impact can be made with the overall limited resources dedicated to health security and preparedness. The BTRP metrics framework will be used as a tool to determine how to best implement key recommendations from NAS and measure their impacts.

To build upon the momentum established during implementation of BTRP's metrics framework and revised annual management life cycle, a critical next step is to implement a more robust technology application for gathering, analyzing, and visualizing metrics data that is easily understood by all stakeholders. Moreover, the effort to harmonize metrics between BTRP, DTRA counterparts, and interagency partners operating in health security must continue in order to have a common operating picture of partner country progress. Lastly, the metrics framework needs to be further developed to measure regional progress and assess sustainment of engagements that have met capability goals where partner countries have moved into self-sustainment.

## Notes

^*^ Cooperative biological research is an integral supportive element of BTRP's comprehensive program. Research is used as an “enabler” to engage partner country scientists in peaceful and ethical application of the biological sciences with a focus on improved biosurveillance, biosafety, and biosecurity. The BTRP research program supports broader program objectives by integrating sustainment of BTRP capacity-strengthening investments through institutionalizing best practices, and resourcing and exercising partner country biorisk management and biosurveillance programs.^5^

^†^ For BTRP, biosurveillance is defined as increasing partner nations' capacities to detect, diagnose, and report disease events quickly, regardless of their point of origin (naturally occurring or manmade), and covers human clinical, veterinary, epidemiology, laboratory science, and health information technology domains.

^‡^ For BTRP, biosafety is defined as enhancing partner nations' abilities to ensure that all operations involving the handling of pathogens are done in a manner that prevents their accidental release or spread.

^§^ For BTRP, biosecurity is defined as securing partner nations' pathogen holdings from internal and external threats to ensure those pathogens are not accessed by parties intent on using them for nefarious purposes.

^**^ Select agents and toxins are defined by the US Department of Health and Human Services and the US Department of Agriculture and identified as especially dangerous due to their abilities to be weaponized by nefarious entities. Select agents and toxins are referred to as especially dangerous pathogens in BTRP.^8^
